# Special Issue ‘Molecular Mechanisms and New Markers of Cancer’—From Static Biomarkers to Context-Aware Precision Oncology

**DOI:** 10.3390/ijms27188085

**Published:** 2026-09-11

**Authors:** Antonio d’Amati

**Affiliations:** 1 Pathology Unit, Department of Laboratory and Hematology Sciences, Fondazione Policlinico Universitario “A. Gemelli” IRCCS, Università Cattolica S. Cuore, 00168 Rome, Italy; damatiantonio@yahoo.it; 2 Department of Medicine and Surgery, LUM University ‘Giuseppe De Gennaro’, 70010 Casamassima, Italy

Cancer molecular medicine has been built on a compelling premise: if the alteration that sustains a tumor can be identified, it can be measured, interpreted, and eventually targeted. The eight contributions collected in this Special Issue show both the power of that premise and the reasons why it must now be refined. Across oncogenic kinases, immune checkpoints, epigenetic regulation, protease control, hormone receptors, transcription factors, and metastatic niches, a common lesson emerges: molecular markers are not static labels. Their biological and clinical meaning depends on the tissue of origin, cellular compartment, disease stage, co-occurring alterations, and the surrounding microenvironment. Precision oncology must therefore move beyond detecting a molecule toward understanding the state and context in which that molecule operates [1,2].

BRAF illustrates this transition particularly well. Mechahougui et al. review how BRAF alterations have become a paradigm for genotype-directed treatment across solid tumors [3]. The success of combined BRAF and MEK inhibition in melanoma established the value of targeting a dominant oncogenic driver, yet the same alteration does not confer uniform sensitivity across malignancies. In colorectal cancer, feedback reactivation through EGFR requires a different combinatorial strategy, while responses in lung cancer and rarer tumors are shaped by distinct co-mutations and tissue-specific signaling networks. Resistance may arise through renewed MAPK activity, activation of PI3K–AKT signaling, or microenvironmental adaptation. Thus, even a highly actionable mutation such as BRAF V600E is not a complete therapeutic instruction by itself, but rather a molecular coordinate that must be interpreted within an organ-specific biological map [4].

The importance of context is equally evident at the immune interface. In ovarian cancer, Borzyszkowska et al. evaluated the tissue expression of LAG-3 and TIM-3 in 58 patients and found frequent expression of both checkpoints, as well as an association between their expression levels [5]. Relationships with age, body mass index, histological category, grade, stage, and menopausal status suggest that immune marker interpretation cannot be separated from clinicopathological variables. These preliminary findings require validation in larger, clinically annotated cohorts, but they support the study of LAG-3 and TIM-3 not only as biomarkers of immune state but also as potential therapeutic targets, including in rational combinations with established checkpoint blockade, antiangiogenic agents, or PARP inhibition. Importantly, co-expression may be more informative than either marker alone because it may capture coordinated immune exhaustion rather than isolated protein positivity [6].

A complementary therapeutic concept is the simultaneous engagement of two tumor-associated targets: pairing a growth factor receptor such as EGFR, HER2, or VEGFR2 with an immunosuppressive molecule such as PD-L1 could improve tumor selectivity while supporting antitumor immunity. Such combinatorial antibody strategies are translationally attractive, although their selectivity, optimal dosing, target density requirements, and safety require rigorous preclinical and clinical testing. This principle emphasizes that useful markers often reside in combinations and spatial patterns rather than in single-analyte measurements [7].

This Special Issue also expands the concept of a cancer marker beyond DNA sequence and cell surface expression. Rembiałkowska et al. synthesize evidence that aberrant DNA methylation, histone modifications, non-coding RNAs, and defective chromatin-remodeling complexes reshape DNA damage recognition and repair [8]. By altering homologous recombination, non-homologous end joining, and base-excision repair, epigenetic dysregulation can generate both vulnerability and resistance. Its reversibility makes the epigenome therapeutically appealing, but its plasticity, heterogeneity, off-target effects, and toxicity complicate durable intervention. This tension is central to biomarker development: a dynamic marker may reveal a therapeutically exploitable state, yet the same dynamism can allow tumor cells to escape. Longitudinal sampling and integrated genomic–epigenomic assessment may therefore be more informative than a single pretreatment snapshot [9,10].

Subcellular localization provides another layer of information. In cancer, cysteine proteases and their endogenous inhibitors may acquire different functional consequences according to their abundance, intracellular distribution, and spatial coordination. These observations suggest that protein levels alone may not adequately describe protease biology, and that localization, interaction with regulators, and preservation of compensatory mechanisms can be equally important. Spatially resolved molecular pathology may therefore identify cancer-specific losses of homeostatic control and reveal candidate therapeutic vulnerabilities [11].

Spatial context becomes still more explicit in metastatic dormancy. Bakir et al. describe how disseminated tumor cells in bone can remain quiescent for years before microenvironmental changes permit relapse [12]. Osteoclast-mediated resorption can release TGF-β and IGF-1; loss of osteoblast-derived quiescence signals can destabilize the endosteal niche; adipocytes can provide metabolic support; and immunosuppressive cells, inflammatory signals, extracellular matrix remodeling, mechanotransduction, and angiogenic switching can collectively enable escape from dormancy. Epigenetic and metabolic reprogramming within disseminated cells further interacts with these niche-derived cues. In this setting, the most clinically consequential marker may not be a fixed property of the tumor cell, but a transition state produced by reciprocal signaling between tumor and host. Detecting or preventing that transition could open an interception window before overt metastatic progression [13].

Estrogen signaling further reinforces the need to replace binary classifications with dynamic models. In gynecological cancers, biological effects may depend on receptor-subtype balance, isoform diversity, subcellular localization, epigenetic regulation, stromal communication, and non-genomic signaling. Loss of epithelial ERalpha does not necessarily indicate loss of estrogen dependence, because signaling may persist through stromal receptors, alternative isoforms, ERbeta, or GPER1. Estrogen-receptor status should therefore be understood as a network state rather than a simple positive or negative result [14,15].

FOXO transcription factors reach a related conclusion. They can enforce cell cycle arrest, apoptosis, stress responses, and metabolic homeostasis, yet under other conditions they may support adaptation, survival, immune regulation, and treatment resistance. Their tumor-suppressive or tumor-promoting behavior varies with biological context and is controlled by post-translational modifications and nucleocytoplasmic trafficking. Therapeutic modulation of FOXO consequently requires temporal and tissue-specific precision; localization and regulatory state may be as important as expression [16].

Taken together, these studies suggest several priorities for the next generation of cancer markers. Biomarkers should be evaluated as multidimensional signatures that combine molecular identity with abundance, localization, co-expression, pathway activity, and microenvironmental context. Validation should be performed in adequately powered, clinically annotated cohorts and should address analytical reproducibility, spatial heterogeneity, and change over time. Mechanistic studies remain indispensable, because association alone cannot establish whether a marker is a driver, a compensatory response, or a bystander. Finally, biomarker development and therapeutic development should proceed together: the most valuable marker is often one that identifies a biological dependency, predicts the need for a rational combination, or reveals an early transition that can be intercepted [17].

Artificial intelligence (AI) offers a practical route from static measurements to context-aware biomarkers. AI-driven multimodal tumor phenotyping can integrate digital histopathology, radiology, spatial data, multi-omics, and longitudinal clinical information to identify patterns that are not captured by any single modality [18,19]. Such approaches may also strengthen AI-driven biomarker validation by testing reproducibility across institutions, platforms, and patient populations and by evaluating whether a candidate adds clinically meaningful information beyond established variables. However, computational performance cannot replace biological and clinical assessment. Models should explicitly examine the context-dependent meaning of each biomarker in relation to tissue of origin, cellular compartment, disease stage, and the surrounding microenvironment, while remaining interpretable enough to generate testable mechanistic hypotheses. Once analytically and clinically validated, integrated AI-derived signatures could support adaptive trial designs in which several biomarkers are considered simultaneously and prespecified interim analyses guide cohort enrichment, treatment allocation, or discontinuation of ineffective arms [20]. Their ultimate value will nevertheless depend on implementation. Complex signatures must be translated, whenever feasible, into robust, rapid, and affordable point-of-care assays or decision-support tools that can operate within routine pathology and oncology workflows. Prospective evaluation, external validation, transparent reporting, attention to bias, data governance, and equitable access will be essential for this progression.

The contributions to this Special Issue span diverse tumor types and experimental perspectives, but they converge on a unified view of precision oncology: cancer is governed by adaptive networks rather than isolated lesions. BRAF activity is conditioned by tissue-specific circuitry; immune checkpoints acquire meaning through co-expression and cellular context; epigenetic states reshape DNA repair; protease regulation depends on intracellular organization; dormant cells are awakened by their niche; and estrogen receptors and FOXO factors can change function across space and time. Translating these insights into patient benefit will require integrated pathology, longitudinal molecular profiling, functional validation, and carefully designed combination strategies. The future of cancer biomarkers lies not merely in asking what is present, but where it is present, when it is active, with which partners it interacts, and which vulnerability that state creates.

## Data Availability

No new data were created or analyzed in this study. Data sharing is not applicable to this article.

## References

[1] Hanahan D. (2022). Hallmarks of Cancer: New Dimensions. Cancer Discov..

[2] Dagogo-Jack I., Shaw A.T. (2018). Tumour Heterogeneity and Resistance to Cancer Therapies. Nat. Rev. Clin. Oncol..

[3] Mechahougui H., Gutmans J., Gouasmi R., Smekens L., Friedlaender A. (2025). BRAF Targeting Across Solid Tumors: Molecular Aspects and Clinical Applications. Int. J. Mol. Sci..

[4] Flaherty K.T., Infante J.R., Daud A., Gonzalez R., Kefford R.F., Sosman J., Hamid O., Schuchter L., Cebon J., Ibrahim H. (2012). Combined BRAF and MEK Inhibition in Melanoma with BRAF V600 Mutations. N. Engl. J. Med..

[5] Borzyszkowska D., Kozłowski M., Golara A., Piotrowska K., Brodowska A., Brodowski J., Bojar I., Kempinska-Podhorodecka A., Cymbaluk-Płoska A. (2025). Association of Tissue Expression of LAG-3 and TIM-3 with Clinical Features in Ovarian Cancer. Int. J. Mol. Sci..

[6] Anderson A.C., Joller N., Kuchroo V.K. (2016). Lag-3, Tim-3, and TIGIT: Co-Inhibitory Receptors with Specialized Functions in Immune Regulation. Immunity.

[7] Labrijn A.F., Janmaat M.L., Reichert J.M., Parren P.W.H.I. (2019). Bispecific Antibodies: A Mechanistic Review of the Pipeline. Nat. Rev. Drug Discov..

[8] Rembiałkowska N., Rekiel K., Urbanowicz P., Mamala M., Marczuk K., Wojtaszek M., Żywica M., Radzevičiūtė-Valčiukė E., Novickij V., Kulbacka J. (2025). Epigenetic Dysregulation in Cancer: Implications for Gene Expression and DNA Repair-Associated Pathways. Int. J. Mol. Sci..

[9] Dawson M.A. (2017). The Cancer Epigenome: Concepts, Challenges, and Therapeutic Opportunities. Science.

[10] Lord C.J., Ashworth A. (2012). The DNA Damage Response and Cancer Therapy. Nature.

[11] Olson O.C., Joyce J.A. (2015). Cysteine Cathepsin Proteases: Regulators of Cancer Progression and Therapeutic Response. Nat. Rev. Cancer.

[12] Bakir M., Dabaliz A., Dawalibi A., Mohammad K.S. (2025). Microenvironmental and Molecular Pathways Driving Dormancy Escape in Bone Metastases. Int. J. Mol. Sci..

[13] Aguirre-Ghiso J.A. (2007). Models, Mechanisms and Clinical Evidence for Cancer Dormancy. Nat. Rev. Cancer.

[14] Arnal J.-F., Lenfant F., Metivier R., Flouriot G., Henrion D., Adlanmerini M., Fontaine C., Gourdy P., Chambon P., Katzenellenbogen B. (2017). Membrane and Nuclear Estrogen Receptor Alpha Actions: From Tissue Specificity to Medical Implications. Physiol. Rev..

[15] Deroo B.J., Korach K.S. (2006). Estrogen Receptors and Human Disease. J. Clin. Investig..

[16] Eijkelenboom A., Burgering B.M.T. (2013). FOXOs: Signalling Integrators for Homeostasis Maintenance. Nat. Rev. Mol. Cell Biol..

[17] McShane L.M., Altman D.G., Sauerbrei W., Taube S.E., Gion M., Clark G.M. (2005). Reporting Recommendations for Tumor Marker Prognostic Studies (REMARK). J. Natl. Cancer Inst..

[18] Boehm K.M., Khosravi P., Vanguri R., Gao J., Shah S.P. (2022). Harnessing Multimodal Data Integration to Advance Precision Oncology. Nat. Rev. Cancer.

[19] Shmatko A., Ghaffari Laleh N., Gerstung M., Kather J.N. (2022). Artificial Intelligence in Histopathology: Enhancing Cancer Research and Clinical Oncology. Nat. Cancer.

[20] U.S. Food and Drug Administration (2019). Adaptive Designs for Clinical Trials of Drugs and Biologics: Guidance for Industry.

